# On the Role of the Excitation/Inhibition Balance of Homeostatic Artificial Neural Networks

**DOI:** 10.3390/e23121681

**Published:** 2021-12-14

**Authors:** Maximilian Brütt, Christian Kaernbach

**Affiliations:** Department of Psychology, Kiel University, 24118 Kiel, Germany; mdpi@kaernbach.de

**Keywords:** artificial neural network, plasticity, homeostasis, self-organization, bifurcation

## Abstract

Homeostatic models of artificial neural networks have been developed to explain the self-organization of a stable dynamical connectivity between the neurons of the net. These models are typically two-population models, with excitatory and inhibitory cells. In these models, connectivity is a means to regulate cell activity, and in consequence, intracellular calcium levels towards a desired target level. The excitation/inhibition (E/I) balance is usually set to 80:20, a value characteristic for cortical cell distributions. We study the behavior of these homeostatic models outside of the physiological range of the E/I balance, and we find a pronounced bifurcation at about the physiological value of this balance. Lower inhibition values lead to sparsely connected networks. At a certain threshold value, the neurons develop a reasonably connected network that can fulfill the homeostasis criteria in a stable way. Beyond the threshold, the behavior of the artificial neural network changes drastically, with failing homeostasis and in consequence with an exploding number of connections. While the exact value of the balance at the bifurcation point is subject to the parameters of the model, the existence of this bifurcation might explain the stability of a certain E/I balance across a wide range of biological neural networks. Assuming that this class of models describes the self-organization of biological network connectivity reasonably realistically, the omnipresent physiological balance might represent a case of self-organized criticality in order to obtain a good connectivity while allowing for a stable intracellular calcium homeostasis.

## 1. Introduction

How do neural networks self-organize so as to build up interesting structures with complex topologies that are capable of processing and storing huge amounts of information that all, even the simplest organisms, have to deal with? In real life, there is no mastermind telling a set of neurons how to connect to each other. Nonetheless, biological neural networks manage to develop a connectivity that is suitable for the tasks imposed by nature. Especially in the mammal cortex, nature seems to have chosen networks with both excitatory and inhibitory neurons to tackle these tasks. At an allotment of about 20–30% [1], inhibitory neurons play an important role in the functioning of the cortex which is yet to be fully understood, though recent years have brought an influx of research on this topic [2,3,4].

Butz and van Ooyen [5] have described a set of rules for homeostasis of the intracellular calcium level of individual neurons. Here, the calcium level is a measure of time-averaged electrical activity of the given neuron [6]. Each neuron will try to homeostatically achieve its set optimal calcium level and by this impetus grow potential docking points of synapse connection, so-called synaptic elements. Synapses will grow between these elements and therefore build connectivity. Merely by specifying these growth rules and giving neurons the ability to build synapses between these elements, neurons are able to dynamically evolve into a complex network, thus following the principles of self-organization regarding their connectivity [7]. We were interested to see how this integration and specifically the balance of excitatory-to-inhibitory neurons in a network would interact with the internal homeostasis rules and affect the number of synapses finally built and the stability of the emanating structure. We present our findings for these questions as well as some results for the role of stimulation input, and we discuss a possible extension of this model and the relevance of our findings to the omnipresent E/I balance in biological networks.

## 2. Methods

For our simulations we employed the NEST simulator with its PyNEST interface [8], and all simulations were executed in Python. We used leaky integrate-and-fire (LIF) neurons and homeostasis parameters based on the parameters proposed by Diaz-Pier et al. [9]. The neurons’ circuit equation can be described as follows:

(1) $$\frac{dV}{dt}=\frac{-\left(V\left(t\right)-V_{rest}\right)}{\tau }+\frac{1}{C}I\left(t\right)$$

where *V*(*t*) denotes the current membrane potential. $V_{rest}=-70\;\mathrm{mV}$ is the resting potential of the neuron. *I*(*t*) is the input current of the neuron. It comprises the internal excitatory, internal inhibitory and external (background) current. The background input has a postsynaptic potential (PSP) with an amplitude of 0.11 mV. Internal currents have PSPs with amplitudes of 1.0 mV for excitatory and −1.0 mV for inhibitory currents. The membrane time constant was τ=10 ms. The membrane capacitance is C=250 pF. The neurons’ spiking threshold was −55 mV. Excitatory and inhibitory neurons in our simulations did not differ in these parameters, but did in their growth curves and type of permitted synaptic elements, as described in the following subsection. Our networks were relatively simple, two-population networks consisting of 100 neurons with varying E/I balance and no spatial relationships between them. Time resolution of the simulations was set to $dt_{sim}=0.1\;\mathrm{ms}$.

### 2.1. Homeostasis Rules

We implemented homeostasis following an algorithm proposed by Butz and van Ooyen [5], and modified in later publications [9,10]. This algorithm can be described by a simple algorithm:

First, the electrical activity of the network, and more specifically, each individual neuron, is determined. This encompasses the processing of membrane potentials, spiking behavior and, in turn, change of the intracellular calcium concentrations of the partaking neurons. The calcium level is changed by a fixed amount β=0.001 (arbitrary units of calcium concentration) with each action potential sent. It decays with $\tau_{Ca}=10,000$ ms. This step happens at every simulation time step $dt_{sim}$, as defined above. Given these parameters, the intracellular calcium update can be expressed as follows:

(2) $$\frac{dCa}{dt}=\{\begin{array}{cc} -Ca\left(t\right)/\tau +\beta , & \mathrm{if}\;\mathrm{the}\;\mathrm{neurons}\;\mathrm{fires}\\ -Ca\left(t\right)/\tau , & \mathrm{else} \end{array}$$

**Figure 1:**
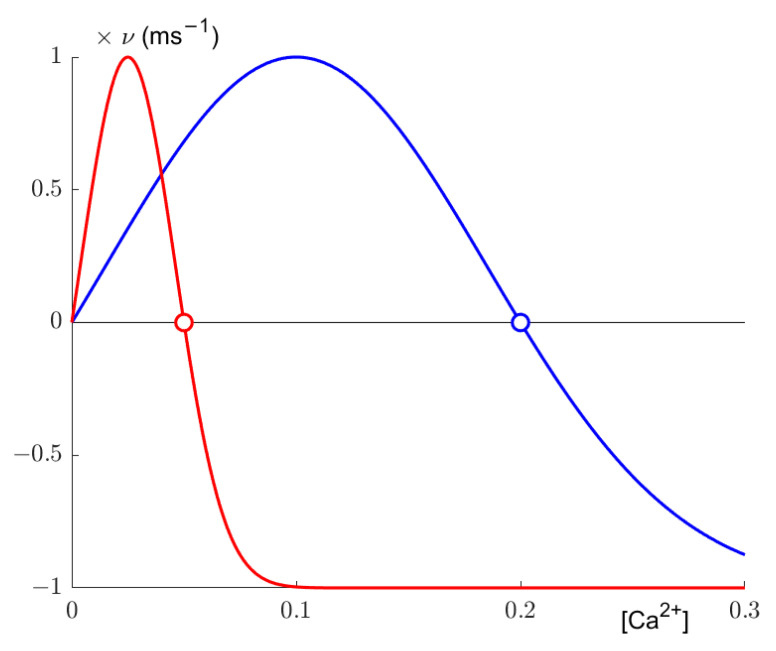
Growth curve for the excitatory (red) and inhibitory (blue) neurons in our simulations. Growth of synaptic elements for an individual neuron is based in the intracellular calcium concentration of the neuron. The neuron will orientate itself towards the set point ε. For most of our simulations, set points were $\varepsilon_{e}=0.05$ and $\varepsilon_{i}=0.2$ (arbitrary units of calcium concentration). The growth rate was ν=0.0001 synaptic elements per ms. The actual number of synaptic elements follows as $z_{t+1}=z_{t}+\lfloor Z\rfloor$ where $z_{t}$ is the number of specific synaptic elements for a given point in time and Z is the sum of synaptic changes since the last growth or deletion for that specific neuron.

Secondly, synaptic elements are updated. According to a Gaussian growth curve (Figure 1), the number of axonal and dendritic elements changes based on the calcium concentration determined in the previous step. The growth curve is defined by its low and high set points η and ε and its growth rate ν. Following the later implementation of Diaz-Pier et al. [9], we set η=0, so our growth curves effectively only have a high set point. The resulting growth curve, as implemented by Diaz-Pier et al. and utilized by us, can be expressed as:

(3) $$\frac{dz}{dt}=\nu \;\left(2\exp\left(-\frac{Ca\left(t\right)-\epsilon }{\frac{\epsilon }{2\sqrt{ln2}}}\right)-1\right),$$

where *z* is the number of synaptic elements of a given type. The set points determine where growth reaches the homeostatic equilibrium, as synaptic elements are deleted if activity is too high, resulting in lesser activity, and synaptic elements are grown if activity is too low, thus potentially increasing the internal calcium concentration. Additionally, following the aforementioned implementation, we defined one growth curve each for excitatory and inhibitory neuron. Excitatory neurons were allowed to grow axonal elements of the excitatory variety only, and inhibitory neurons could grow only inhibitory axonal elements. Dendritic elements were not restricted.

Thirdly, synapses are formed. A synapse can be formed if two compatible synaptic elements are vacant, i.e., no synapse is connected to them yet. For each free axonal element, an unoccupied dendritic element of the same type (excitatory or inhibitory) is chosen. Each of the unoccupied elements can be chosen with the same probability. Additionally in this step, synapses are pruned by deletion of synaptic elements. The deletion of an occupied element, as per the rules described in step two, leads to the deletion of the associated synapse. The previously connected synaptic element at the other end, however, remains intact. Everything in this step happens at a second time interval, the connectivity update step $dt_{conn}$. Generally, $dt_{sim}$ is much smaller than $dt_{conn}$ to reflect the relatively slow changes in synaptic structure in physiology.

### 2.2. Simulation Parameters

We aimed to simplify the parameter and rule set used for the simulations as much as possible, to impose only a small set of constraints on the otherwise freely behaving network. All simulations were performed for populations with N=100 neurons, with the E/I balance ranging from 100:0 to 50:50. There was no starting topology specified in any way and there were no spatial relationships between our neurons. Our simulation parameters were derived from the aforementioned work on simulations of homeostatic plasticity. The values for, e.g., the intracellular calcium set levels were based on these parameters and have no origin in biology. The parameters, however, could be scaled, as long as their internal relationships are preserved, should the need arise to reflect findings in cell physiology.

For external stimulation we used NEST’s Poisson spike generator. We varied the rate of the Poisson spike generator from 9000 to 11,000 Hz. Simulation time resolution was set to $dt_{sim}=0.1\;\mathrm{ms}$, with connectivity updates being computed every $dt_{conn}=1000\;\mathrm{ms}$. This ratio corresponds to biological processes, albeit the structural changes are expected to be far slower, taking up to several days [5]. We chose to implement these faster update times to not unnecessarily slow the simulations down. Most simulations ran for a simulated time of 1000 s (for exceptions, see below). This was sufficient to judge whether the simulation reached homeostatic equilibrium or not.

### 2.3. Assessing Stability

To assess whether the simulation reached equilibrium and could be considered stable, we calculated an envelope with width ±0.02 over the target calcium levels for excitatory and inhibitory neurons separately and determined the percentage of data points in the mean calcium concentration outside of this envelope. We considered a simulation to be stable when the percentage of data points outside the envelope was below 25%. This relatively liberal criterion was set up in order to allow for an initial transient phase before achieving the target levels. For stable networks, the total number of synapses was considered as a measure of connectivity.

## 3. Results

**Figure 2:**
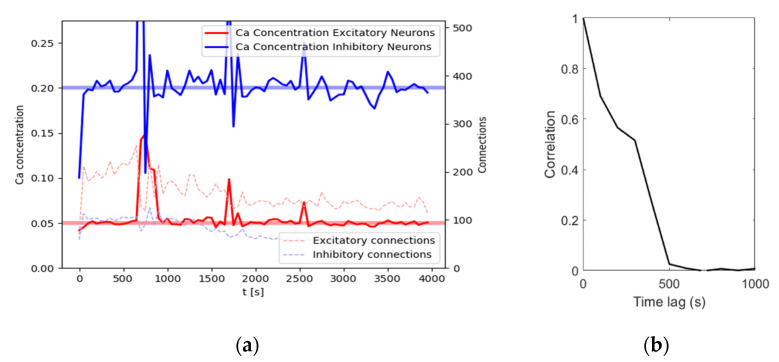
Results for a network consisting of 80 excitatory and 20 inhibitory neurons for a simulation time of 4000 s. (**a**) Intracellular calcium levels (left axis) averaged over all neurons of the respective subpopulation (E/I). The number of connections (right axis) is the sum of all connected axonal elements of the respective subpopulations. Data points are given for $dt_{rec}=50\;s$ to avoid cluttering; (**b**) Time-lagged correlation for the connectivity matrix of the network at time slice t=1000 s with the following connectivity matrices in steps of 100 s. The number of connections stays similar while the correlation with previous states decreases, indicating a dynamic equilibrium.

We replicated the findings of Diaz-Pier et al. [9] with our first parameter set of 80 % excitatory neurons and 20 % inhibitory neurons largely using the authors’ homeostasis parameters with a network of N=100 neurons in contrast to the authors’ net size of 1000 individual cells. For an illustration of achieved calcium levels and connections, see Figure 2a. With an input noise rate of r=10,000 Hz, the network reaches homeostatic equilibrium quickly after around 100 s of simulation time, with the calcium levels oscillating around the target levels for the remainder of this simulation. Connectivity shows a slightly different dynamic. At about 500 s, connection density reaches a maximum. After that, connection density decreases for a while, reaching a stable level after about 2000 s. In this example, the network reaches a size of 114 excitatory connections and 44 inhibitory connections at 4000 s. While the number of connections does not change much, the connection matrix changes dynamically as the connectivity continuously renews itself. This is evidenced by a constantly decreasing correlation with the following connectivity matrices (Figure 2b). In other words, after another 1000 s there will be about the same number of connections, but these connections will not be the same anymore.

Explorative testing has shown that a network of the same composition but sizes of N=20 neurons or N=1000 neurons shows comparable behavior with larger or smaller variance, respectively. As we aimed to show homeostatic behavior at a comparatively small, yet not innately volatile level, we settled for the N = 100 shown here as our simulation network size. We will refer to this configuration, with a simulation time shortened to 1000 s for computation cost, as the base rule set throughout the rest of this work.

### E/I Balance, Noise Level and Stability

As outlined above, we were interested in the interplay of the homeostasis algorithm and the E/I balance of the network. We varied the E/I balance of the network in increments of 5, starting at 100:0 (a fully excitatory network). To compensate for potential losses in activity when diminishing the excitatory share of neurons in the network, we additionally and independently varied the input rate of spike trains given by the Poisson generator from 9000 Hz to 11,000 Hz. We repeated each simulation five times with different random seeds. Stability was assessed by looking at the mean calcium levels of the excitatory and inhibitory subpopulations and comparing them to the respective target level at each time step (see Section 2.3). A certain parameter combination was considered to yield stable results when it proved stable in all five repetitions. In the case of stable parameter combinations, we determined the connectivity by averaging the final connectivity after 1000 s across the five repetitions.

**Figure 3:**
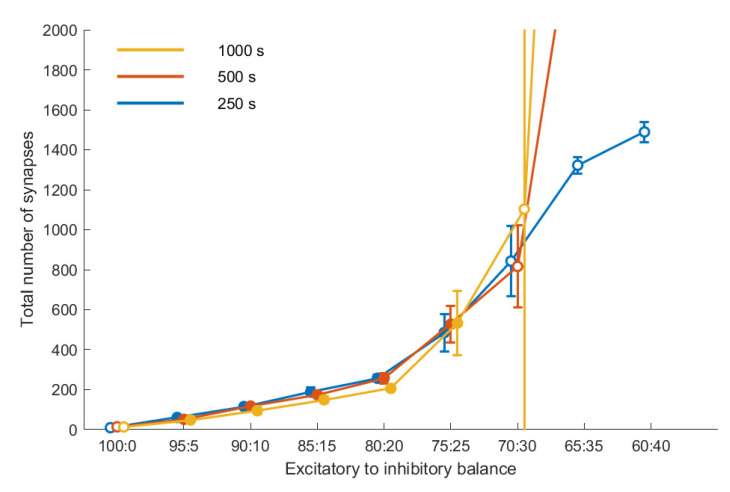
Development of the connectivity in our networks after 250, 500 and 1000 s. All results are averaged over five runs. The error bars indicate the standard deviations. Markers filled white indicate runs where the homeostasis did not reach equilibrium according to our metric.

Figure 3 shows the connectivity for a subset of our data as a function of the E/I balance for three points in time, for a fixed noise level (10,000 Hz). With increasing share of inhibition, connectivity increases. Up to an E/I balance of 75:25, the connectivity reached after 250 s remains stable up to the final duration of the simulation of 1000 s, i.e., there is no significant difference between the three curves. Moreover, connectivity is reproducible; there is little variation across the five repetitions, as indicated by the relatively small error bars. This coincides with the evaluation of these E/I balance values as leading to stable homeostasis.

E/I balance values beyond that threshold value do not lead to a stable connectivity. This becomes obvious in the deviation of the three curves, showing that the connectivity differs at the three time points with a tendency for the later to be larger, and in addition it shows in the larger error bars for these E/I balance values, indicating a lower degree of reproducibility. The outcome of our stability analysis (Section 2.3) is coded by the fill color of the symbols in Figure 3: Simulations that did not fulfill the stability criterion are indicated by symbols with a white fill.

**Figure 4:**
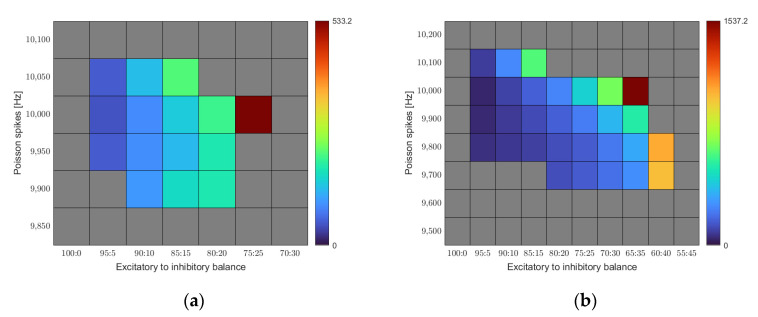
Mean synapse counts for different E/I balances and different noise input levels. All results shown are averaged over five runs. Cells shaded grey are where at least one of the five runs did not achieve stability. (**a**) The calcium set levels for excitatory and inhibitory neurons were set to $\varepsilon_{e}=0.05$ and $\varepsilon_{i}=0.2,\;\mathrm{respectively}$; (**b**) Here, the calcium set level for inhibitory neurons was lowered to $\varepsilon_{i}=0.1$.

Figure 4a represents the outcome of our simulations as a function of the E/I balance and noise level. We find a region of stability constrained in both dimensions. Only a certain range of input noise leads to stable results. Low Poisson spike rates quickly lead to the activity dying off. The minimum required input rate to reach equilibrium in our base rule set was 9900 Hz. On the other hand, the network quickly succumbed to overstimulation when driven with a Poisson spike rate above 10,050 Hz. For the E/I balance, we find increasing connectivity with increasing share of the inhibition up to a certain point. Beyond that point, homeostasis no longer reaches its equilibrium and connectivity increases without bounds. The optimal combination for the given set of calcium target levels is an E/I balance of 75:25 at a noise level of 10,000 Hz.

Figure 4b shows a similar map for a different set of calcium target levels. While we kept the target level for the excitatory cells constant at 0.05, we chose a lower target level (0.1 instead of 0.2) for the inhibitory neurons this time.

For this set of target levels, the region of stability shifts. A larger range of noise levels leads to stable homeostasis, and the threshold point of the E/I balance gets shifted towards higher inhibition values. It becomes obvious that the region of stability depends on the exact settings of the model parameters.

## 4. Discussion

We studied the connectivity of simple two-population neural networks with different E/I balances and different stimulation inputs. The networks were solely driven by internal homeostasis rules of the intracellular calcium level of the individual neurons. The neurons did not receive any meaningful input; instead, input was provided by applying noise to the dendrites of the neurons. It is not trivial that artificial neural networks developing under these rules can tell us anything interesting about biological networks. In this perspective, we find it very interesting that certain constraints on the balance of excitatory and inhibitory cells coincide with physiological findings.

It appears that the E/I balance is a parameter that plays an important role to achieving homeostasis as well as concerning the connectivity that can be built in a stable manner. Purely excitatory networks will in our class of models not achieve a stable homeostasis. A small share of inhibitory neurons is sufficient to grant a stable network, even if at rather small connectivity values.

Adding inhibition up to a certain point will lead to an increasing number of synapses, still keeping the homeostatic target values. The resulting networks show a stable connectivity in the sense that the number of connections fluctuates slightly around stable values. The connectivity is, however, not stable in the sense that synapses, once established, will have a stable existence. In such a dynamically stable network, synapses are continually built up and pruned, with an average lifetime of some hundred seconds. Continuous changes in structure are typically found in developing biological networks which have yet to build up a structure. Our artificial networks are, in a sense, in this state of self-discovery, as they start without a single synapse between them. They also share the globally random input that a developing network without functioning sensory receptors would be exposed to, such as under sensory deprivation, essentially facilitating random, spontaneous synapse formation. Remedy for and augmentation of this behavior could be a more “meaningful” input, as we discuss below.

At a certain point, the homeostatic equilibrium can no longer be granted. With calcium levels well below the target values, the networks develop an exceeding amount of synapses way beyond anything that can be found in biology, and without reaching a point of saturation with a stable number of synapses. In biology, such a greedily expanding network would prove disadvantageous. Exhausting energy for building up countless synapses that might not even increase performance of the network would be a behavior to avoid. Energy resources and spatial constraints would limit the otherwise ever-increasing connectivity. One could modify the model so as to include such constraints. In fact, biological systems seem to respect this threshold point and stay close to it but clearly below. On the threshold we found, the network exists in a state in which it reaches its calcium target values and avoids connectivity excesses. Any state induced by E/I balances with inhibitory shares below this point leads to a less connected network; states induced by a higher inhibitory share do not reach their homeostatic equilibrium at all and build up excessively many synaptic connections. It seems hence not necessary to add constraints to the model to fence off outcomes that are anyway not to be expected in nature.

We did not find any topological structure in our networks. The networks in our simulations show a homogeneous distribution of synapses, so that the synapse building probability is the only relevant structural parameter needed to describe the network. This is not surprising, as we studied networks developing without any meaningful input. We are aware that much is missing to generate very interesting topologies. Adding meaningful input to a network developing under the rules of homeostasis would be an interesting step to analyze the impact of inhibition in the development of two-population networks. Coherent input, for example in a spatially structured way, could emulate naturally plausible stimulation and therefore nudge the connectivity dynamics more towards behavior observed in vivo. As discussed above, networks in vivo exhibit relative synaptic stability. We found our networks in both dendritic and axonal elements to fluctuate, thus showing behavior previously found in developing networks [11] or under sensory deprivation [12,13]. After a certain point in ontogenetic development, network connectivity appears to settle and synaptic structure is largely stable, with a small share still in a state of fluctuation [14]. Studies predominantly in mice have shown this late synaptic fluctuation to be linked with sensory experiences [15,16]. These experiences could be modeled as spatially coherent input, following the representation of input, e.g., in the retina.

Spatially structured input could also give rise to structure in the network. With implementation of spike-timing-dependent plasticity, we would expect to see an increase in connectivity within ensembles that are spatially close and therefore stimulated together. The resulting structures would not only be biologically plausible, but also interesting to study with our methods of E/I balance variation and homeostatic plasticity.

The interplay of excitatory and inhibitory neurons has been discussed as an important player for the emergence of critical phases in juvenile brains with enhanced possibilities for plastic changes of the connectivity [10] (p. 183). In our simulations, we set the number of excitatory and inhibitory neurons in each single simulation. It would be an interesting extension of this class of models if the share of inhibitory neurons would itself be part of the homeostatically regulated parameters. This idea is not far from physiology, as changing chloride concentration in the development of a neuron can indeed change its role in a circuit, therefore changing the E/I balance at large [17].

In summary, we find that the E/I balance plays an important role in developing artificial networks with a reasonable connectivity. Starting from a purely excitatory network, adding inhibition will increase the connectivity up to a certain point. A certain E/I balance seems to be optimal for a not-too-sparse but still stable connectivity. Beyond that value, adding further inhibition will corrupt the homeostatic algorithms, leading to connectivity excesses that have not been observed in nature. This finding holds for a certain range of noise levels and for varying calcium target levels.

It is highly interesting to note that for certain parameter settings the threshold value of the E/I balance found in our simulations corresponds closely to the E/I balance found over a wide range of biological networks. While in our simulations the E/I balance was set, in nature, networks develop by growth and have to self-organize so as to find a good E/I balance. In further studies, we want to explore further terrains of the parameter space in order to verify whether our finding generalizes to other parameter settings. If it proves to be a stable feature of homeostatic artificial neural networks, it puts a finger on a possible case of self-organized criticality, and would call for models that can regulate the E/I balance by themselves.

## Data Availability

The data presented in this study are available on request from the corresponding author.
